# Novel Gerota-edge-sling technique facilitates retroperitoneal robot-assisted partial nephrectomy: a comparative study

**DOI:** 10.1186/s12894-022-01079-4

**Published:** 2022-08-20

**Authors:** Wei Chen, Qixiang Fang, Haomin Ren, Lei Ma, Jin Zeng, Shangshu Ding, Dapeng Wu

**Affiliations:** 1 Department of Urology, The First Affiliated Hospital of Xi’an Jiaotong University, Xi’an, 710061 People’s Republic of China; 2 Department of Anesthesiology and Perioperative Medicine, The First Affiliated Hospital of Xi’an Jiaotong University, Xi’an, People’s Republic of China; 3 Department of Urology, Shaanxi Provincial People’s Hospital, Xi’an, People’s Republic of China

**Keywords:** Kidney neoplasms, Partial nephrectomy, Robotic surgical procedures, Retroperitoneal approach, Surgical technique

## Abstract

**Background:**

Retroperitoneal robotic partial nephrectomy is markedly restricted by limited space and visual field. We introduced a novel Gerota-edge-sling (GES) technique with self-designed traction devices to overcome these defects by attaching Gerota fascia to abdominal wall, and comparatively evaluated its utilization with routine technique.

**Methods:**

A retrospective analysis was performed for consecutive patients who underwent routine (control group) or GES assisted (GES group) retroperitoneal robotic partial nephrectomy for localized renal tumors in our hospital between March 2018 and June 2020. Clinical data of perioperative outcomes and complications were collected and compared. Comparison of outcomes between anterior versus posterior tumor subgroups was also conducted. Linear regression analysis was used to define the relationship between dissection time and perinephric fat status in each group.

**Results:**

Totally 103 patients were included, 48 in control and 55 in GES group respectively. All the procedures were completed successfully without conversion or positive surgical margin. GES group had significantly decreased console time (91 ± 36 min vs. 117 ± 41 min, *p* < 0.01) and dissection time (67 ± 35 min vs. 93 ± 38 min, *p* < 0.01) than control, while ischemia time, blood loss, and nephrometry score comparable between them. No major postoperative complications occurred. Dissection time of GES group was notably shorter than that of control in both anterior/posterior subgroups. Only in control group, dissection time was positively associated with perinephric fat status.

**Conclusions:**

The GES technique acting as an adjunct to robotic arms with space-sparing feature, notably improves surgical exposure and facilitates dissection in retroperitoneal partial nephrectomy, while having great feasibility, efficacy and safety.

**Supplementary Information:**

The online version contains supplementary material available at 10.1186/s12894-022-01079-4.

## Background

Partial nephrectomy (PN) is recognized as the gold standard for management of small renal tumors because of its equivalent oncological outcome and better renal function preservation compared with radical nephrectomy [1]. The advent of robotic system substantially expands the range of minimally invasive PN by its exclusive features which allow more precise and steady manipulation than traditional laparoscopic surgery [2, 3].

Robot-assisted partial nephrectomy (RAPN) can be performed through either transperitoneal or retroperitoneal approach, and the optimized one remains debated. Theoretically, retroperitoneal approach harbors multiple advantages, including avoiding bowel mobilization, directly accessing renal hilum, constraining blood or urine to retroperitoneal space, and furthermore, potentially decreasing operative time, blood loss, and postoperative complications [4]. However, actually transperitoneal approach has more often been used than retroperitoneal in RAPN. The restrained application of retroperitoneal approach could be partially attributed to the obvious defect of small working space greatly limited by Gerota fascia [5]. In addition, even with robotic platform continuing to evolve, poor vision owing to the curtain effect of Gerota fascia always necessitates the use of a fourth arm to aid in traction/countertraction, especially for anterior renal tumors, raising challenges to avoid collisions.

To overcome these defects, here we introduced a novel GES technique to assist retroperitoneal RAPN (rRAPN) for both anterior and posterior renal tumors by pulling Gerota fascia attaching to abdominal wall to expand retroperitoneal space, and comparatively evaluated its perioperative outcomes and complications with routine three-arm technique.

## Methods

### Patients selection

This study was conducted in accordance with the ethical standards of the institutional and/or national research committee, and approved by the ethics committee of The First Affiliated Hospital of Xi’an Jiaotong University. Informed consent was collected for all patients.

The medical records of consecutive patients who underwent routine (control group) or GES assisted (GES group) rRAPN for preoperative imaging showing unilateral asymptomatic contrast-enhancing renal tumors were retrospectively reviewed (March 2018–June 2020). Tumors with the maximal diameter ≥ 10 cm, anterior hilar tumors as well as ones at Brodel line were excluded. No patient had a history of ipsilateral retroperitoneal surgical intervention. CT angiography was performed to identify the renal artery branches to guide intraoperative clamping.

### Data collection

All the procedures were performed by the same surgeon (D.P.W.). The obtained data included demographic information, tumor characteristics and perioperative outcomes. Console time, dissection time (DT) (defined as the time lapse after trocar insertion until hilum clamping), warm ischemia time (WIT), estimated blood loss (EBL), positive surgical margin (PSM), transfusion requirement, complications classified by the Clavien–Dindo system, and postoperative length of stay (LOS) were recorded as perioperative outcomes. Anatomical characteristics of renal tumors and perinephric fat status were described using R.E.N.A.L. (Radius, exophytic/endophytic, nearness, anterior/posterior, location) nephrometry score and Mayo Adhesive Probability (MAP) score respectively [6, 7]. For R.E.N.A.L. systems, tumors were further categorized into low (4–6), moderate (7–9), or high (10–12) group for complexity stratification.

Follow-up was conducted every 3 months for the first 2 years. Follow-up visits comprising physical examination, serum chemistry evaluation and imaging studies were performed according to institutional protocols and at the discretion of physician when clinically indicated.

### Surgical and GES techniques

The previously described technique of rRAPN with three robotic arms and single assistant port near anterior axillary line was used for all cases by the da Vinci Si system™ (Intuitive Surgical Inc., Sunnyvale, CA, USA) [8]. Briefly, after Gerota fascia incised, dissection was carried along psoas muscle to elevate kidney and perinephric fat to stretch the hilum. And then the renal artery was identified and skeletonized.

**Fig. 1:**
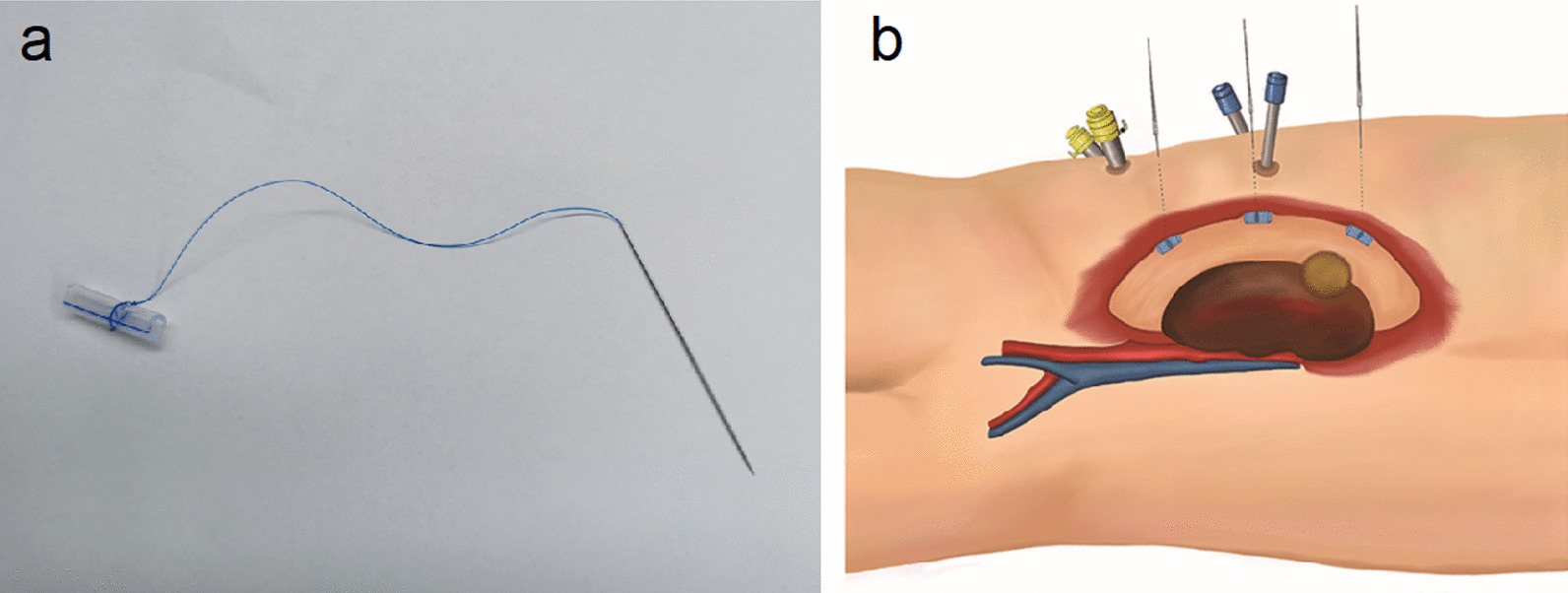
The diagrams of GES technique. **a** Composition of the traction device. Straight needle with polypropylene suture fastened with silica gel tube. **b** GES technique scheme

**Fig. 2:**
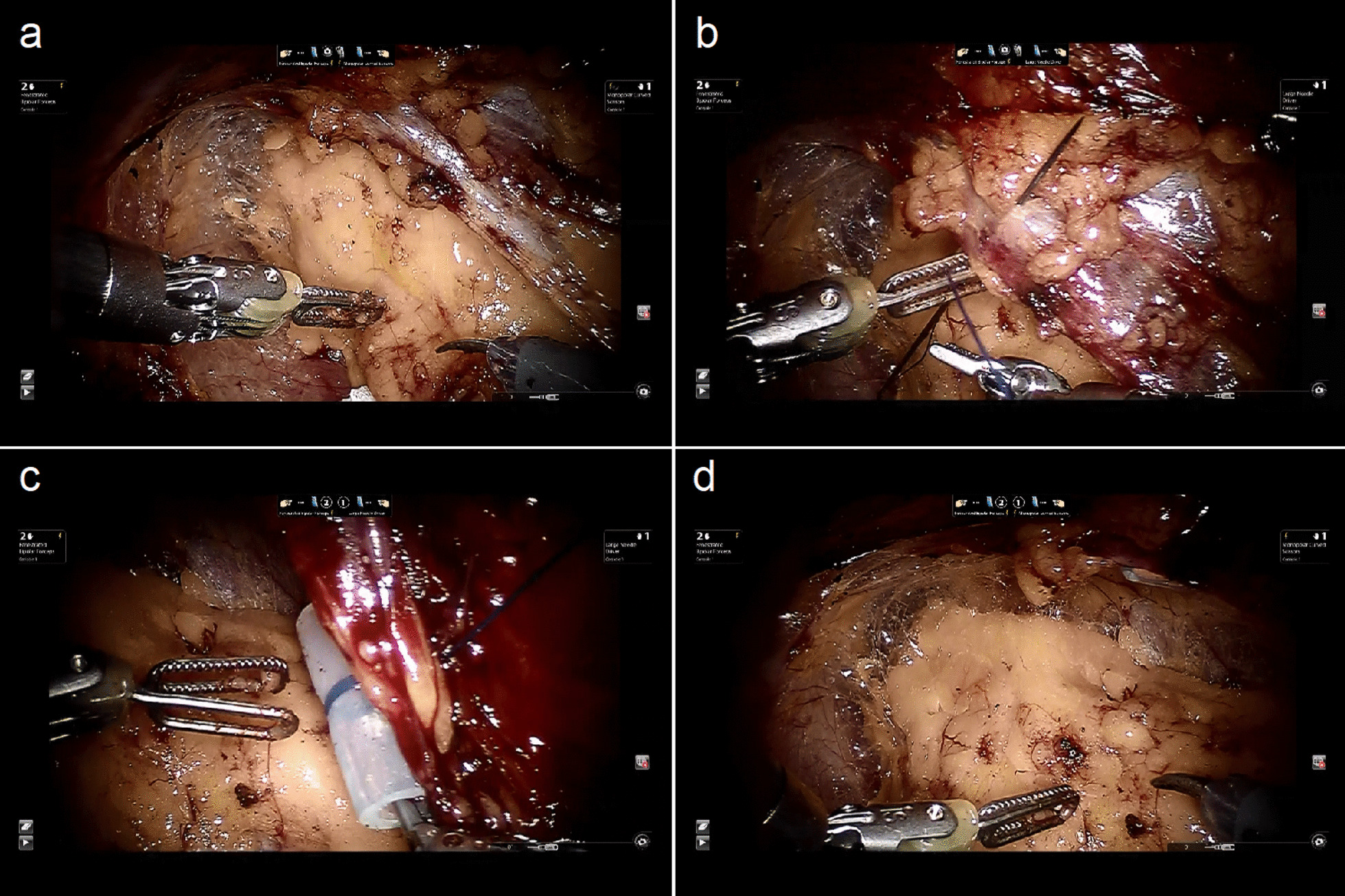
The procedure of setting GES device and the effect. **a** Surgical vision before using GES technique. **b** Straight needle penetrating through the edge of Gerota fascia to abdominal wall. **c** Silica gel tube pulled to support the attachment of Gerota fascia to abdominal wall. **d** Surgical vision after using GES technique

The procedure then continued routinely in control group. But in GES group, one or two self-designed traction devices were set after initial dissection of perinephric fat to unveil the cutting edge of Gerota fascia. The traction device was composed of a 65 mm long straight needle with a 10 cm long 2-0 polypropylene suture (Ethicon Prolene Polypropylene Suture, D9080), which was fastened to a 2 cm long silica gel tube (Fig. 1a). Three potential sling points on the edge could be selected, corresponding to renal hilum, upper and lower pole of kidney (Fig. 1b). Single sling point adjacent to tumor location was always used. If still not satisfying, two points would be selected. The traction device was introduced into retroperitoneal space by the assistant, and then the needle was punctured vertically through certain sling point and abdominal wall to extracorporeal side (Fig. 2b). After pulling out the needle, the gel tube tightly anchored Gerota fascia to abdominal wall by stretching the suture with its external end fixed by forceps (Fig. 2c). Then the dissection continued. Tumor excision and renorrhaphy were performed under warm ischemia. Generally, the double-layer running suture with 3-0 and 2-0 V-Loc (Covidien, Mansfield, MA, USA) was utilized for renorrhaphy, but for superficial cortical tumors, single-layer suture was preferred. The specimen was extracted within an entrapment bag. At last, all the traction devices were retrieved. Detailed information of the procedure with GES technique was shown in an additional movie clip (Additional file 1). A postoperative drain was placed in the retroperitoneal space. Intraoperative frozen section analysis would be performed if necessary.

### Statistical analysis

Continuous variables were compared with the student’s t-test and reported as means with standard deviations (SD). Categorical variables were compared with chi-square and Fisher exact test, ordered categorical variables were compared with Mann–Whitney U-test, all of them were reported as numbers and percentages. Unpaired t-test was used for nephrometry scores, operative data, and postoperative LOS. Linear regression analysis was used to fit data to a model that defines a relationship between DT and MAP score. All statistical analyses were performed using Statistical Package for the Social Sciences version 20 (SPSS Inc., Chicago, IL), with *p* < 0.05 indicating statistical significance.

## Results

Overall, 103 patients were included in the study, 48 in control group and 55 in GES group respectively. A single renal tumor presented in each patient, except one patient in GES group harboring two ipsilateral lesions (see Additional file 1). By using GES technique, the edge of Gerota fascia was firmly attached to abdominal wall and the curtain effect was eliminated. Moreover, retroperitoneal space was largely expanded with excellent view of kidney achieved (Fig. 2a, d). All the procedures were carried out successfully, and no need of conversion to open surgery or radical nephrectomy.

Table 1 shows the comparison of clinical characteristics between these two groups. While demographic data and tumor features comparable, GES group had significantly decreased console time (91 ± 36 min vs. 117 ± 41 min, *p* < 0.01) and dissection time (67 ± 35 min vs. 93 ± 38 min, *p* < 0.01) than control. Despite shorter WIT in GES group, but the difference did not reach statistical significance (*p* = 0.46).

**Table 1:** Patient demographics, operative and tumor characteristics, stratified according to surgical techniques

	Control group	GES group	*p*
	N = 48	N = 55	
Mean ± SD age (ys)	54.7 ± 12.7	53.3 ± 12.6	0.57
Gender			0.22
No. male (%)	39 (81.3)	39 (70.9)	
No. female (%)	9 (18.7)	16 (29.1)	
No. laterality (%)			0.53
R	30 (62.5)	31 (56.4)	
L	18 (37.5)	24 (43.6)	
No. Anterior/posterior tumors (%)			0.57
Anterior	25 (52.1)	26 (46.4)	
Posterior	23 (47.9)	30 (53.6)	
Mean ± SD BMI (kg/m2)	25.2 ± 2.8	24.3 ± 3.7	0.18
Mean ± SD ASA score	2.1 ± 0.5	2.0 ± 0.5	0.29
Mean ± SD Charlson comorbidity index	1.9 ± 1.6	2.0 ± 1.2	0.76
Mean ± SD Console time (mins)	117 ± 41	91 ± 36	< 0.01
Mean ± SD DT (mins)	93 ± 38	67 ± 35	< 0.01
Mean ± SD WIT (mins)	21.3 ± 5.0	20.6 ± 4.2	0.46
Mean ± SD EBL (ml)	81 ± 92	74 ± 44	0.61
Mean ± SD Length of stay after operation (days)	4.3 ± 0.7	4.3 ± 0.9	0.44
No. Clavien–Dindo complication (%)			0.82
None	43 (89.6)	48 (87.3)	
Grade I	3 (6.3)	5 (9.1)	
Grade II	2 (4.1)	2 (3.6)	
Mean ± SD tumor diameter (mm)	35 ± 11	38 ± 10	0.29
Mean ± SD R.E.N.A.L. score	7.3 ± 1.8	7.4 ± 1.8	0.73
No. R.E.N.A.L. complexity (%)			0.85
Low	14 (29.2)	16 (29.1)	
Moderate	30 (62.5)	33 (60.0)	
High	4 (8.3)	6 (10.9)	
Mean ± SD MAP score	1.9 ± 1.6	2.2 ± 1.3	0.26
No. histology (%)			0.86
Clear cell	44 (91.7)	50 (90.9)	
Papillary	1 (2.1)	0 (0)	
Chromophobe	2 (4.1)	3 (5.5)	
Other malignant	1 (2.1)	2 (3.6)	
No. pathological stage (%)			0.86
T1a	31 (64.6)	33 (60.0)	
T1b	16 (33.3)	22 (40.0)	
T2a	1 (2.1)	0 (0)	

*L* Left; *R* Right; *No* Number; *SD* Standard deviation

Either R.E.N.A.L. score for quantitating tumor complexity or MAP score for predicting perinephric fat status did not differ between the two groups. No major complications occurred in the patients. There were 5 and 7 cases of minor (Grade I and II) postoperative complications recorded in control and GES group respectively, demonstrating the similar complication rates (*p* = 0.82). No case of PSM was found. During the median period of 15-month follow-up, no local recurrence or distal metastasis had occurred.

Based on the comparison of the entire groups, further evaluation of perioperative features in both anterior and posterior tumor subgroups was conducted (Table 2). 25 (52.1%) tumors located at anterior side in control and 26 (46.4%) in GES group. For anterior tumors, both console time and DT in GES group were significantly shorter than those in control group (*p* < 0.01 respectively). However, for posterior tumors, only DT in the GES group notably decreased (*p* = 0.04), while these tumors possessing larger diameter than that of control (40 ± 10 mm vs. 32 ± 11 mm, *p* = 0.01).

**Table 2:** Subgroup comparison of perioperative characteristics between the two groups

	Control group	GES group	*p*
*Anterior tumors*			
Mean ± SD BMI (kg/m2)	25.1 ± 2.5	24.9 ± 3.4	0.80
Mean ± SD Console time (mins)	125 ± 43	92 ± 36	< 0.01
Mean ± SD DT (mins)	100 ± 41	69 ± 35	< 0.01
Mean ± SD WIT (mins)	22.0 ± 4.9	20.3 ± 4.3	0.18
Mean ± SD EBL (ml)	69 ± 48	66 ± 33	0.77
Mean ± SD tumor diameter (mm)	38 ± 12	36 ± 10	0.39
Mean ± SD R.E.N.A.L. score	7.6 ± 1.7	7.5 ± 1.6	0.83
Mean ± SD MAP score	2.1 ± 1.6	2.2 ± 1.4	0.86
*Posterior tumors*			
Mean ± SD BMI (kg/m2)	25.2 ± 3.2	23.7 ± 3.9	0.14
Mean ± SD Console time (mins)	107 ± 36	90 ± 37	0.12
Mean ± SD DT (mins)	84 ± 34	66 ± 35	0.04
Mean ± SD WIT (mins)	20.5 ± 5.2	20.9 ± 4.1	0.73
Mean ± SD EBL (ml)	93 ± 123	71 ± 52	0.62
Mean ± SD tumor diameter (mm)	32 ± 11	40 ± 10	0.01
Mean ± SD R.E.N.A.L. score	7.1 ± 1.9	7.3 ± 1.9	0.63
Mean ± SD MAP score	1.7 ± 1.6	2.2 ± 1.3	0.17

*SD* Standard deviation

**Fig. 3:**
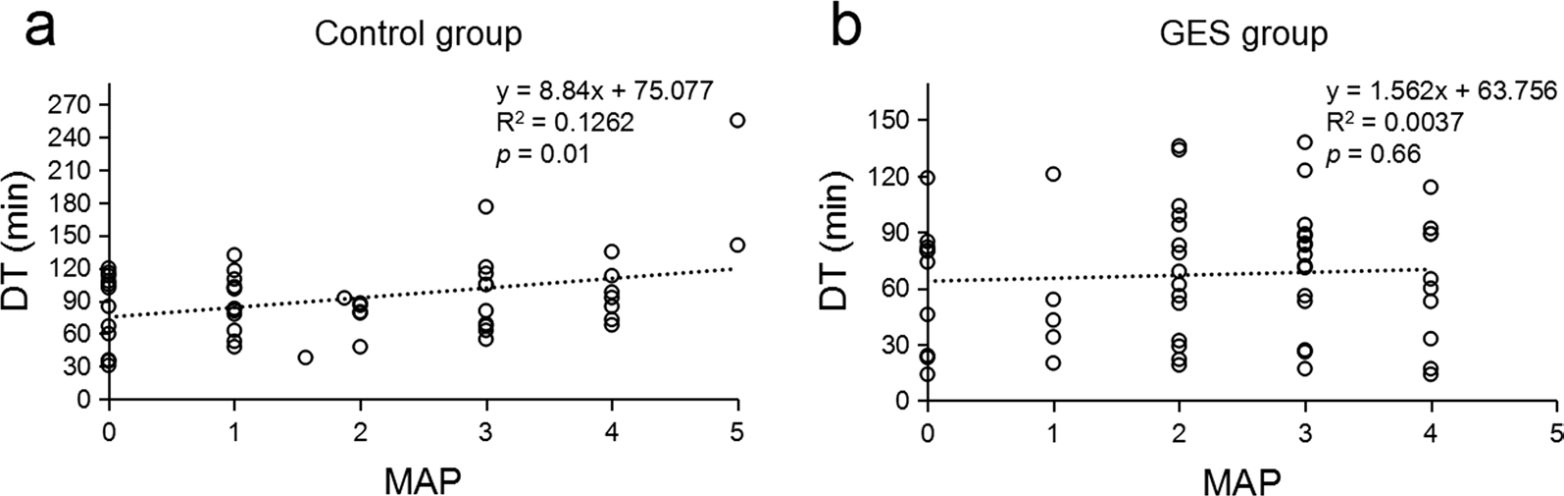
Linear regressions between MAP score and dissection time (DT) of control **a** /GES **b** group respectively. Each circle in the figure represents an individual patient

The distribution of DT according to MAP score was shown in Fig. 3. Remarkable prolonged DT was observed in control group as MAP score increased. Linear regression was employed to predict dissection duration based on MAP score for each group. A regression equation was found for control group (*p* = 0.01), whereas not for GES group (*p* = 0.66).

## Discussion

It has been well demonstrated retroperitoneal laparoscopic PN has comparable oncological and functional outcomes with transperitoneal approach [4, 9, 10]. Although the da Vinci system possesses high level of flexibility and efficiency of manipulation, rRAPN is still greatly restricted by the inherent limitations of small working space and poor vision, which could not be simply transformed with the evolution of robotic platforms [2, 4, 11]. Except for posterior tumors, retroperitoneal approach has been used much less than transperitoneal one, reflected by the essentially low ratio of anterior tumors in previous studies. However, recently two large multi-institutional studies vigorously rejected the hypothesis of superior operative outcomes decided by the choice of surgical approach based on tumor’s location [12, 13]. Nevertheless, the optimal strategy effectively improving the experience of rRAPN to magnify its exclusive advantages needs further investigation.

In rRAPN, tissue traction to facilitate exposure and visualization has always been accomplished by placement of additional devices, either a fourth robotic arm or second assistant port [8, 14]. But for the setting of such devices, peritoneum needs to be further swept towards medial plane, potentially increasing the incidence of peritoneal leak. Although the da Vinci Xi system has helped reduce collisions more commonly encountered with the Si system, careful manipulation should be taken to proceed in the limited space which accommodates five shafts concurrently. Recently introduced magnetic tool may be another option, but it would embezzle the space with probably compromised coupling effect when thick abdominal wall encountered [15].

In this study, we introduced the self-designed traction device that was convenient to be made with ordinary materials and evaluated the safety and efficacy of its utilization as GES technique to assist rRAPN. With the aid of GES technique, the visualization and space of retroperitoneal approach could be enormously and steadily improved, which mimics the effect of additional device. This feature is especially important for dealing with large or complex renal tumors. Based on the similar preoperative patient characteristics, including BMI, American Society of Anesthesiology (ASA) score, Charlson Comorbidity Index (CCI), R.E.N.A.L. and MAP score, GES technique showed more efficiency in console time and DT than control, while sharing analogous postoperative complication profiles. In our cohort, almost half of the tumors in both groups were located at anterior portion of kidney. And the subgroup analyses revealed GES technique could improve DT for both anterior and posterior tumors. This can be explained probably that for anterior tumors, GES substantially expanded the ventral perinephric space, which liberated the assistant from traction, and for posterior ones, GES pulled kidney toward ventral side together with the fat capsule to facilitate exposure. In addition, a potential advantage of GES technique relied on its space-sparing feature while functioning without collision-raising shaft. Meanwhile, no transfusions, need of conversion or major (Clavein ≥ III) complications occurred in any patient. The results has far reaching implications, as it provides surgeons an alternative or adjunct for the fourth-arm or transperitoneal approach, helping delineate the optimal surgical route and provide preoperative medical consultations.

To date, this is the first report to describe GES-assisted rRAPN. We demonstrated the technique was feasible without significant modification of the original procedures. There were no absolute contraindications or limitations regarding the application of GES. Kim et al. reported they exposed the anterior perinephric space by stitching the edge of Gerota fascia, followed by pulling out with Endo Close™ trocar-site closing device, which potentially had the risk of impairing peritoneum [16]. Although the edge of Gerota fascia was running sutured, enough support could not be provided only by thin suture, leading to potential inadequate exposure. However, GES technique was carried out from inside to outside with thick gel tube, and above defects were avoided effectively. There was no rupture of peritoneum during dissection in our cohort, which could be ascribed to the limited peeling of peritoneum from abdominal wall and additional tension for peritoneum provided by GES.

We used the mainstream R.E.N.A.L. nephrometry system to quantify tumor complexity and evaluate PN risk [6]. Typically, complex tumor involves laborious dissection, resection and renorrhaphy, leading to prolonged operative time, WIT and excessive EBL. Although R.E.N.A.L. score includes the variable indicating anterior/posterior position of tumor, which is potentially less problematic for open but more for laparoscopic surgery, the variable remains non-quantitated and fails to account for risk stratification. To better elucidate the role of GES technique for tumors on different sides, we reclassified them into anterior/posterior subgroups. In both subgroups, GES technique notably accelerated the process of dissecting tumors with comparable R.E.N.A.L. scores. Furthermore, no significant difference of R.E.N.A.L. scores was observed between the anterior and posterior tumors in each group. While sharing similar console time and DT in GES group, anterior tumors in control group obligated remarkably longer console time and DT than the posterior ones (data not shown), which was in accordance with the previous report [16]. This implies GES technique tremendously facilitated the manipulation of rRAPN, substantially extending retroperitoneal approach to anterior tumors under its assistance. Given the background of R.E.N.A.L. system, further development of nephrometry specifically for robotic surgery or certain approach is needed.

Moreover, adherent perinephric fat (APF) was recognized as an important non-tumor-related factor complicating PN by increasing the difficulty of kidney mobilization and tumor isolation [17]. MAP score was an image-based system to quantify the possibility and severity of APF [7]. Our study revealed higher MAP score predicted more DT in control group with linear regression model, whereas not in GES group. It may be explained by the fact that GES technique liberated the assistant from the dilemma of traction or suction especially when APF encountered. With the aid of GES, the effort was concentrated on dissection, consequently equalizing difficulties reflected by MAP scores.

Altogether, this study made a novel technical improvement in RAPN with retroperitoneal approach. The findings clearly showed its advantages for the treatment of renal tumors with acceptable short-term outcomes. GES technique which only took a few minutes to be established possesses great efficacy and safety, which could promote rRAPN as an alternative strategy for the treatment of anterior tumors [18].

A few limitations exist in the current study. The retrospective design and small sample size of our cohort allows sources of potential selection bias. Owing to the short follow-up time, long-term oncological outcomes are still under further evaluation. Although the relatively outdated Si system still being extensively used, due to continuous improvement of robotic platform, the efficacy of GES technique needs to be externally validated in contemporary systems. Additionally, to further demonstrate its usefulness, a prospectively random-designed study is crucial in the future.

## Conclusions

The GES technique facilitates rRAPN for both anterior and posterior renal tumors by overcoming surgical obstacles, such as limited working space and annoying sticky perinephric fat. The technique expands the domain of rRAPN, while having great feasibility, efficacy and safety, as an adjunct to the routine technique.

## Supplementary Information

**Additional file 1**: A movie clip showing the detailed information of rRAPN with GES technique. https://figshare.com/articles/media/Novel_Gerota-edge-sling_technique_facilitates_retroperitoneal_robot-assisted_partial_nephrectomy/20071652.

## Data Availability

The datasets used and analyzed during the current study are available from the corresponding author on reasonable request.

## Abbreviations

RAPN: Robot-assisted partial nephrectomy
rRAPN: Retroperitoneal robot-assisted partial nephrectomy
GES: Gerota-edge-sling
DT: Dissection time
PN: Partial nephrectomy
WIT: Warm ischemia time
EBL: Estimated blood loss
PSM: Positive surgical margin
LOS: Length of stay
ASA: American society of anesthesiology
CCI: Charlson comorbidity index
